# Neddylation Facilitates the Antiviral Response in Zebrafish

**DOI:** 10.3389/fimmu.2019.01432

**Published:** 2019-06-25

**Authors:** Guangqing Yu, Xing Liu, Jinhua Tang, Chenxi Xu, Gang Ouyang, Wuhan Xiao

**Affiliations:** ^1^State Key Laboratory of Freshwater Ecology and Biotechnology, Institute of Hydrobiology, Chinese Academy of Sciences, Wuhan, China; ^2^The Key Laboratory of Aquaculture Disease Control, Ministry of Agriculture, Wuhan, China; ^3^The Key Laboratory of Aquatic Biodiversity and Conservation, Institute of Hydrobiology, Chinese Academy of Sciences, Wuhan, China; ^4^University of Chinese Academy of Sciences, Beijing, China; ^5^The Innovation Academy of Seed Design, Chinese Academy of Sciences, Wuhan, China

**Keywords:** *nedd8*, zebrafish, MLN4924, antiviral response, innate immunity, neddylation, SVCV

## Abstract

Neddylation is a type of post-translational protein modifications, in which neural precursor cell expressed developmentally downregulated protein 8 (NEDD8) is covalently conjugated to the lysine residues of target substrates. The best characterized principal substrates of neddylation are the cullin-RING ligases (CRLs). In addition, neddylation also modifies non-cullin proteins to affect gene regulation, cell survival, organ development, and stress response. However, the role of neddylation in antiviral innate immunity remain largely unknown. Here, we found that when neddylation was blocked by the NEDD8 activating enzyme E1 (NAE) inhibitor, MLN4924, the cellular and organismal antiviral response was suppressed. Moreover, the disruption of *nedd8* increased the sensitivity of zebrafish to SVCV infection. Further assays indicated that blocking or silencing neddylation significantly downregulated key antiviral genes after poly (I:C) stimulation or SVCV infection, but dramatically increased SVCV replication. Neddylation of Irf3 and Irf7 was readily detected, but not of Mda5, Mavs, and Tbk1. Thus, our results not only demonstrated that neddylation facilitated the antiviral response *in vitro* and *in vivo*, but also revealed a novel role of *nedd8* in antiviral innate immunity.

## Introduction

Neddylation is a type of post-translational protein modifications in which neural precursor cell expressed developmentally downregulated protein 8 (NEDD8) is covalently conjugated to the lysine residues of target substrates (1). Like ubiquitination, neddylation is triggered by the sequential actions of NEDD8 activating enzyme E1 (NAE), NEDD8-conjugation enzyme E2 and NEDD8-E3 ligase (1). Neddylation is a reversible modifications; protein deneddylation is performed by deneddylases, such as DEN1/SENP8 (2, 3). The best characterized principal substrates of Neddylation are the cullin-RING ligases (CRLs), in the E3 ubiquitin ligase family (4). However, neddylation also modifies non-cullin targets, regulating substrate protein activity, stability, and subcellular localization (5–8). Functionally, neddylation is critical for gene regulation, cell survival, organ development, and the stress response (6, 7). Dysregulation of neddylation is associated with disease pathogenesis (9–11).

The role of protein neddylation in immunological regulation has received increasing attentions (12–14). The inhibition of neddylation leads to the suppression of LPS-induced pro-inflammatory cytokine production in macrophage cells (15). Neddylation regulates T-cell function by targeting Shc and Erk signaling (16), and is also required for HSV-1-induced early phase IFN-beta production (17). In addition, neddylation of Myd88 or BCA3 indirectly downregulates NF-κB signaling (18, 19). Recently, it has been shown that blocking the neddylation pathway suppresses influenza virus replication and the pro-inflammatory response (20). Finally, neddylation enhances CD4^+^ T cell-mediated protective immunity against–blood stage *Plasmodium* infection (12). Interestingly, MLN4924, an inhibitor of NAE, inhibits TLR3/4 and retinoic acid-inducible gene I-induced IFN-β expression by preventing IRF3 binding to the IFN-β promoter, with a neddylation-independent manner (21).

Although neddylation may be involved in multiple immune responses, it is still largely unclear whether neddylation in response to pathogenic infection benefits or damages the host. This uncertainty remains because suitable assays, particularly *in vivo* animal models are lacking. Zebrafish (*Danio rerio*) is a model organism that has been used for studies of the antiviral response *in vivo* (22, 23). Here, we used a cell culture system and a zebrafish model to show that blocking neddylation suppressed the antiviral immune response and that disruption of *nedd8* reduced the ability of zebrafish to combat viral infection. Our results thus demonstrated that neddylation played an important role in facilitating the host antiviral response.

## Materials and Methods

### Cells and Zebrafish

We cultured epithelioma papulosum cyprini (EPC) cells (originally obtained from the American Type Culture Collection, Manassas, VA, USA) in medium 199 (Biological Industries, Cromwell, CT, USA) supplemented with 10% fetal bovine serum (FBS). We cultured zebrafish liver (ZFL) cells (originally obtained from the American Type Culture Collection) in 50% L-15 (Invitrogen, Carlsbad, CA, USA), 35% DMEM-HG (Invitrogen), and 15% Ham's F12 medium (Invitrogen) supplemented with 0.15 g/l sodium bicarbonate (Sigma-Aldrich, St. Louis, MO, USA), 15 mM HEPES (Sigma-Aldrich), and 10% FBS. EPC cells and ZFL cells were maintained at 28°C in a humidified incubator containing 5% CO_2_. HEK293 T cells were maintained at 37°C in a humidified incubator containing 5% CO_2_.

### Viral Infection

We propagated Spring Viremia of Carp Virus (SVCV, an ssRNA virus that causes an important disease affecting cyprinids) in EPC cells until the cytopathic effect (CPE) was complete. We collected the culture medium and stored it at −80°C until use. Viral titers were determined by a 50% tissue culture-infective dose (TCID_50_) assay in EPC cells. The final virus titer was adjusted to ~2 × 10^8^ TCID_50_/ml.

For viral challenge of zebrafish larvae, thirty 3-dpf zebrafish larvae per group in triplicate were challenged for 24 h at 25°C in disposable 60 mm cell culture dishes by immersion in ~2 × 10^8^ TCID_50_/fish SVCV (24). Simultaneously, MLN4924 (1 mM-MLN4924 dissolved in DMSO; 5 μl) or vehicle (DMSO; 5 μl) were added to egg water. After challenge, the remaining fish in each group were transferred to fresh plates containing egg water and monitored every 8 h over a 48 h period to score mortality (25). In addition, for examining gene expression, the total RNA was extracted and quantitative real-time PCR (qPCR) assays were conducted.

For viral challenge of adult zebrafish, 3 mpf adult zebrafish (0.38 ± 0.02 g) were each intraperitoneally (i.p.) injected with 10 μl of SVCV (~2 × 10^8^ TCID_50_/ml) using 10 μl Microliter syringes (Shanghai Gaoge Industry and Trade Co., Ltd., Shanghai, China). Zebrafish i.p. injected with PBS were used as the controls. After viral challenge for 48 h, zebrafish were anesthetized with tricaine methanesulfonate and dissected. The kidneys and spleens were collected and stored at −80°C for further qPCR assays.

### Validation for Injected mRNA

Myc-*nedd8* and GFP were subcloned into Psp64 poly (A) vector (Promega). AmpliCap SP6 High Yield message maker kit (Epicenter) was used for capped mRNA synthesis. Myc-*nedd8* and GFP mRNA were synthesized and injected into zebrafish embryos at one-cell stage (400 pg/per embryo). To confirm expression of injected mRNAs, the embryos injected with Myc-nedd8 mRNA for 3 days were harvested and the expression of Myc-nedd8 was confirmed by Western blot using anti-Myc antibody (9E10, Santa Cruz).

### Generation of nedd8-Null Zebrafish

We disrupt *nedd8* in zebrafish using CRISPR/Cas9 techniques. The primers for detecting mutation are: 5′- AATGTGAATCTCGTTCAGGTGG-3′ and 5′-AGATGTACAGGAACACAACGTG−3′. The *nedd8* mutant was named (*nedd8* ^ihb1227/ihb1227^) (https://zfin.org/ZDB-ALT-180718-1), following zebrafish nomenclature guidelines. To exclude off-targeting effects, we back-crossed *nedd8*-null zebrafish with wild-type (WT) zebrafish (strain AB; no siblings of the heterozygous zebrafish were included). After repeating this back-crossing for five generations, the F6 adult zebrafish carrying the same mutation were used for breeding. Due to the low fecundity of the *nedd8*-null females, we mated *nedd8* ^+/−^ (♀) with *nedd8* ^−/−^ (♂) to obtain *nedd8* ^+/−^ and *nedd8* ^−/−^ larvae for viral infection. To generate WT larvae, we mated the *nedd8* ^+/+^ (♀and ♂) siblings of the *nedd8* ^−/−^ mutant.

Zebrafish were maintained in a re-circulating water system following standard protocols. All experiments with zebrafish were approved by the Institutional Animal Care and Use Committee of Institute of Hydrobiology, Chinese Academy of Sciences (protocol number 2016-018).

### MLN4924 Treatment

We dissolved MLN4924 (1 mM, in DMSO) (Selleckchem., Houston, TX, USA) to medium or egg water and yielded a final MLN4924 concentration of 1 μM. Controls were treated equivalent volumes of DMSO.

### CPE Assay

EPC cells were seeded in 12-well plates overnight and treated with either vehicle (DMSO) or MLN4924 (1 μM) for 24 h, then infected with SVCV at MOI of 1, 10, 100, 1,000 for 2 days. Subsequently, the cells were washed three times with 1 × PBS and fixed with 4% paraformaldehyde for 20 min. The fixed cells were stained with 1% crystal violet.

### Cell Viability Assay

Cell viability was determined by the Cell Counting Kits (CCK-8) (Yeasen, HB171114) following the manufacturer's instructions. Briefly, EPC cells or ZFL cells were seeded into 96-well cell culture plates (approximately 1 × 10^4^ cells/per well) and cultured for 24 h at 28°C. The medium was replaced with fresh medium supplemented with either vehicle (DMSO) or MLN4924 (1 μM), and the cells were inoculated with SVCV (MOI of 10) for 20 h, then added CCK-8 solution. At the time points: 24, 48, 72, 96, 120 h, we measured the cell viability, respectively. The optical density was determined at 450 nm using a microplate reader (Spectra Max® MiniMax^TM^ 300 Imaging Cytometer). All standards and samples were measured by three independent experiments performed in triplicate.

### Plasmid Construction and Neddylation Assay

The open reading frame (ORF) of zebrafish *nedd8* (Gene ID: 368667) was amplified by PCR and then cloned into pCI (Clontech). The cDNAs encoding *mavs* (Gene ID: 562867), *mda5* (Gene ID: 565759), *tbk1*(Gene ID: 692289), *irf3* (Gene ID: 564854), *irf7* (Gene ID: 562867) were subcloned into pCMV-Myc (Clontech).

Neddylation assays were performed as reported previously with some modifications (26). Briefly, HEK293T cells were transfected with the indicated constructs for 16–22 h, and then harvested the cells. The cells were lysed using the lysis buffer (6 M guanidine hydrochloride, 0.1 M Na_2_HPO_4_/NaH_2_PO_4_, 10 mM imidazole and 10 mM mercaptoethanol). Subsequently, the lysates were mixed with Ni^2^-NTA-agarose beads (Qiagen, Valencia, CA) pre-washed with lysis buffer, and rotated at 4°C overnight. The beads were washed three times using washing buffer I (1/5 lysis buffer plus 4/5 washing buffer II (25 mM Tris/HCl (pH 6.8) plus 20 mM imidazole) and washed another 3 times using washing buffer II. The beads were eluted with the sample-loading buffer and analyzed by Western blot assays.

### Western Blot Assay

The following antibodies were used for Western blot assays: anti-glyceraldehyde-3-phosphate dehydrogenase (GAPDH) (Santa Cruz), anti-β-actin (Santa Cruz), anti-Histone H3 (cell signaling technology), anti-nedd8 (ABclone).

HEK293T cells were transfected with different combinations for 24 h, then the cells were lysed in RIPA buffer containing 50 mM Tris (pH 7.4), 1% Nonidet P-40, 0.25% sodium deoxycholate, 1 mM EDTA (pH 8.0), 150 mM NaCl, 1 mM NaF, 1 mM PMSF, 1 mM Na3VO4, and a 1:100 dilution of protease inhibitor mixture (Sigma-Aldrich), After incubation on ice for 1 h, the lysates were centrifuged at 10,000 × g at 4°C for 15 min. The total cell lysate were boiled with 1xSDS sample loading buffer, separated on SDS-PAGE, and transferred to a polyvinylidene difluoride membrane (Millipore). Western blot assay was performed as described previously (22). The Fujifilm LAS4000 mini-luminescent image analyzer was used to image the blots.

### Quantitative Real-Time PCR (qPCR) Analysis

Total RNA was extracted from cells, embryos, and tissues (kidney and spleen) using RNAiso Plus (TaKaRa Bio., Beijing, China), following the manufacturer's instruction. cDNAs were synthesized using the Revert Aid First Strand cDNA Synthesis Kit (Thermo Scientific, Waltham, MA, USA). MonAmp^TM^ SYBR® Green qPCR Mix (high Rox) (Monad Bio., Shanghai, China) was used for quantitative RT-PCR (qPCR) assays. The primers for qPCR assays are listed in Supplemental Table S1. Actb1 (β-actin) was used as an internal control.

### Statistical Analysis

Quantitative real-time PCR, and virus titer data are reported as means ± SEM of three independent experiments, each performed in triplicate. The statistical analysis was performed using GraphPad Prism 5 software using unpaired *t*-test (GraphPad Software). The log-rank test was used to calculate the differences in survival of the different experimental groups. Differences with *P* < 0.05 were considered statistically significant (25).

## Results

### Inhibition of Neddylation via the Addition of MLN4924 Suppressed the Cellular Antiviral Response

**Figure 1:**
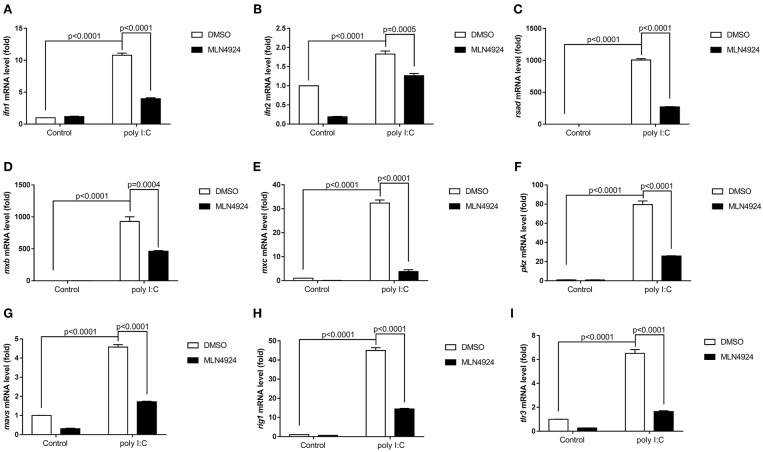
MLN4924 suppresses the expression of IFN and ISGs in ZFL cells after poly (I:C) stimulation. **(A–I)** Treatment of ZFL cells with 1 μM MLN4924 after poly(I:C) stimulation downregulated *ifn1* **(A)**, *ifn2* **(B)**, *rsad* **(C)**, *mxb* **(D)**, *mxc* **(E)**, *pkz* **(F)**, *mavs* **(G)**, *rig1* **(H)**, *tlr3* **(I)**. Additions of the same amount of DMSO were used as controls. Data are shown as mean ± SEM of three independent experiments, each performed in triplicate; the statistical analysis was performed using GraphPad Prism 5 (unpaired *t*-test).

**Figure 2:**
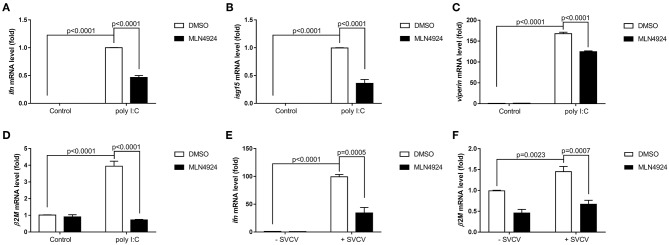
MLN4924 suppresses the expression of key antiviral genes in EPC cells after poly(I:C) stimulation or SVCV infection. **(A–D)** Treatment of EPC cells with 1 μM MLN4924 after poly(I:C) stimulation downregulated *ifn* **(A)**, *isg15* **(B)**, and *viperin* **(C)**, β*2m* **(D)**. **(E,F)** Treatment of EPC cells with 1 μM MLN4924 after SVCV infection downregulated *ifn* **(E)** and β*2m* **(F)**. Data are shown as mean ± SEM of three independent experiments, each performed in triplicate; the statistical analysis was performed using GraphPad Prism 5 (unpaired *t*-test).

As a pharmacological inhibitor of NAE, MLN4924 specifically blocks NEDD8 activation and, consequently, the neddylation pathway (27). To date, most studies examining the role of neddylation in various cellular processes have used MLN4924 (12, 15, 17, 20, 21, 28). To elucidate the role of neddylation in cellular antiviral immunity, we treated cells with MLN4924 and then quantified the expression levels of the key antiviral genes after poly (I:C) treatment, the procedure mimicked a double-strand RNA (dsRNA) virus (22). Compared to the control treatment (DMSO), MLN4924 treatment suppressed the expression of several key antiviral genes (e.g., *ifn1, ifn2, rsad, mxb, mxc, pkz, mavs, rig1*, and *tlr3*) in ZFL cells after poly (I:C) stimulation (Figures 1A–I) (22). MLN4924 treatment also suppressed the expression of key antiviral genes (e.g., *ifn, isg15, viperin*, and β*2m*) in EPC cells after poly (I:C) stimulation, as compared to the control treatment (DMSO) (Figures 2A–D). In addition, MLN4924 treatment suppressed the expression of key antiviral genes (*ifn* and β*2m*) in EPC cells after SVCV infection compared to the control treatment (DMSO) (Figures 2E,F).

**Figure 3:**
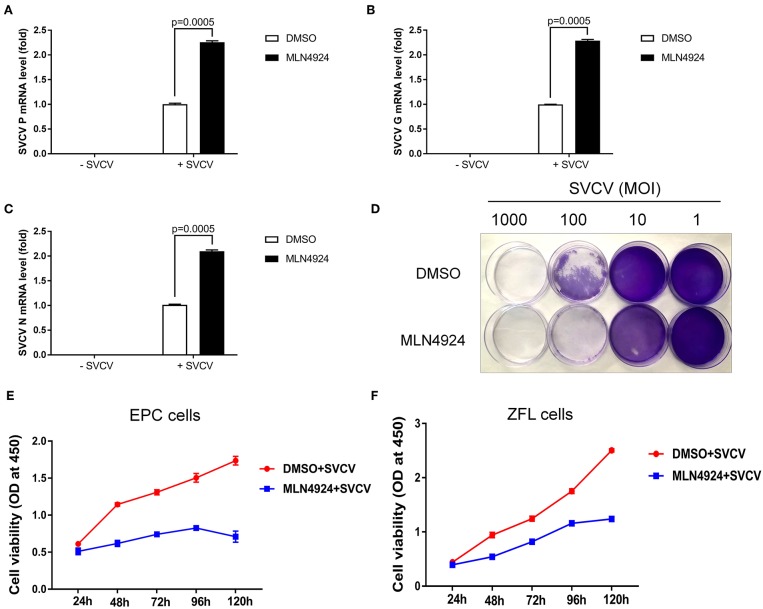
MLN4924 increases SVCV replication in EPC cells. **(A–C)** In EPC cells, MLN4924 treatment after SVCV infection increased the copy number of SVCV-related genes, as compared to vehicle (DMSO)-treated cells. We treated EPC cells with either vehicle (DMSO) or MLN4924 (1 μM) for 24 h, and then infected the cells with SVCV (MOI of 10). After SVCV infection for 24 h, we extracted total RNA and used quantitative real-time PCR (qPCR) assays to determine the mRNA expression levels of the SVCV *P, G*, and *N* genes. **(D)** Treatment with 1 μM MLN4924 after SVCV infection reduced survival in EPC cells. **(E,F)** In EPC cells **(E)** and ZFL cells **(F)**, MLN4924 treatment after SVCV infection inhibited cell proliferation. We treated EPC cells or ZFL cells with either vehicle (DMSO) or MLN4924 (1 μM) for 24 h, and then infected the cells with SVCV (MOI of 10) for 24 h. Cell viability was determined using the Cell Counting Kits at the indicated time points. Data are shown as mean ± SEM of three independent experiments, each performed in triplicate; the statistical analysis was performed using GraphPad Prism 5 (unpaired *t*-test).

Consistently, MLN4924 treatment increased SVCV replication in EPC cells, based on the increased expression of the SVCV *P, G*, and *N* genes, as compared to the control treatment (DMSO) (Figures 3A–C). As expected, CPE assays showed MLN4924 treatment reduced EPC cell survival after SVCV infection, as compared to the control treatment (DMSO) (Figure 3D). In consistent with the CPE assays, MLN4924 treatment inhibited cell proliferation of both EPC cells and ZFL cells after SVCV infection (Figures 3E,F).

Our data thus suggested that the inhibition of neddylation suppressed the cellular antiviral response.

### Inhibition of Neddylation via the Addition of MLN4924 Induced Suppression of the Zebrafish Antiviral Response

**Figure 4:**
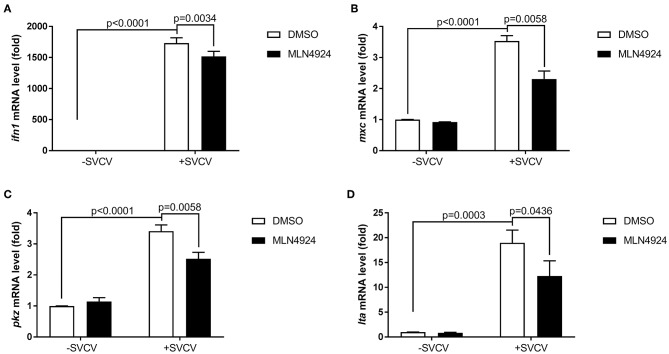
MLN4924 suppresses SVCV-induced activation of key antiviral genes in zebrafish larvae. **(A–D)** The expression levels of *ifn1* **(A)**, *mxc* **(B)**, *pkz* **(C)**, and *lta* **(D)** after SVCV infection were lower in zebrafish larvae treated with MLN4924 as compared to larvae treated with DMSO (control). Zebrafish larvae (three days post-fertilization, dpf) were infected with SVCV (~2 × 10^8^ TCID_50_/ml) after pretreatment with either vehicle (DMSO) or MLN4924 (1 μM) for 24 h. After 24 h incubation, we extracted total RNA from all larvae and used qPCR assays to detect the expression levels of *inf1, mxc, pkz*, and *lta*. Data are shown as mean ± SEM of three independent experiments, each performed in triplicate; the statistical analysis was performed using GraphPad Prism 5 (unpaired *t*-test).

**Figure 5:**
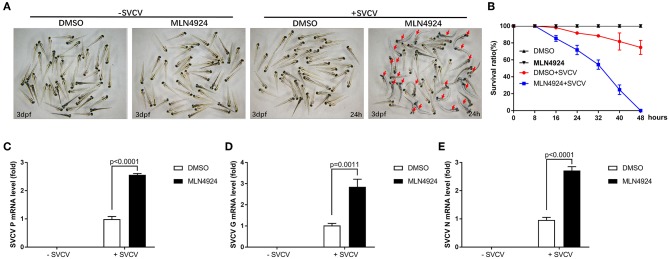
Zebrafish larvae treated with MLN4924 are more sensitive to SVCV infection. **(A)** Representative images of zebrafish larvae (3 dpf), both uninfected and infected with SVCV for 24 h, after treatment with the vehicle (DMSO; the control) or MLN4924 (1 μM). Dead larvae (indicated with red arrows) were characterized by a lack of movement, absence of blood circulation, and bodily degeneration. **(B)** Survival ratios indicated that zebrafish larvae treated with MLN4924 were more sensitive to SVCV infection than were larvae treated with vehicle (DMSO). Zebrafish larvae (3 dpf; *n* = 90 in total) were infected with SVCV (2 × 10^8^ TCID_50_/ml) after pretreatment with either vehicle (DMSO) or MLN4924 (1 μM); this experiment was repeated three times (*n* = 30 for each). We counted the numbers of dead larvae at 8, 16, 24, 32, 40, and 48 h post-infection. **(C–E)** Viral replication was much greater in SVCV-infected zebrafish larvae treated with MLN4924 (1 μM), as compared with the control. Zebrafish larvae were infected with SVCV after pretreatment with either vehicle (DMSO; the control) or MLN4924 (1 μM). After incubation for 24 h, we used qRT-PCR assays to determine the expression levels of the SVCV genes *P* **(C)**, *G* **(D)**, and *N* genes **(E)**. Data are shown as mean ± SEM of three independent experiments, each performed in triplicate; the statistical analysis was performed using GraphPad Prism 5 (unpaired *t*-test).

We examined the role of neddylation during the *in vivo* antiviral response using zebrafish as the model organism. Initially, we checked expression of *inf1* and *pkz* in zebrafish larvae (3 dpf) treated with different dosage of MLN4924 for 24 h and subsequently infected with SVCV for 24 h. 1 μM MLN4924 could suppress both *inf1* and *pkz* expression dramatically (Supplemental Figure S1). Subsequently, we chose 1 μM MLN4924 for treatment of zebrafish larvae. Similar to the results obtained for ZFL and EPC cells, MLN4924 treatment suppressed the expression of key antiviral genes (e.g., *ifn1, mxc, pkz*, and *lta*) after SVCV infection, as compared to the control treatment (DMSO) (Figures 4A–D). Furthermore, MLN4924 treatment decreased the survival rate of zebrafish larvae after SVCV infection, as compared to the control treatment (DMSO) (Figures 5A,B). Dead zebrafish larvae were recognized by lack of movement, absence of blood circulation, and bodily degeneration (Figure 5A). Consistently, SVCV replication, as indicated by the expression levels of the SVCV *P, G*, and *N* genes was significantly higher in MLN4924-treated larvae as compared to the DMSO-treated larvae (Figures 5C–E). Thus, our data suggested that blocking neddylation suppressed antiviral response *in vivo*.

### Nedd8 Facilitated the Antiviral Response in Zebrafish

**Figure 6:**
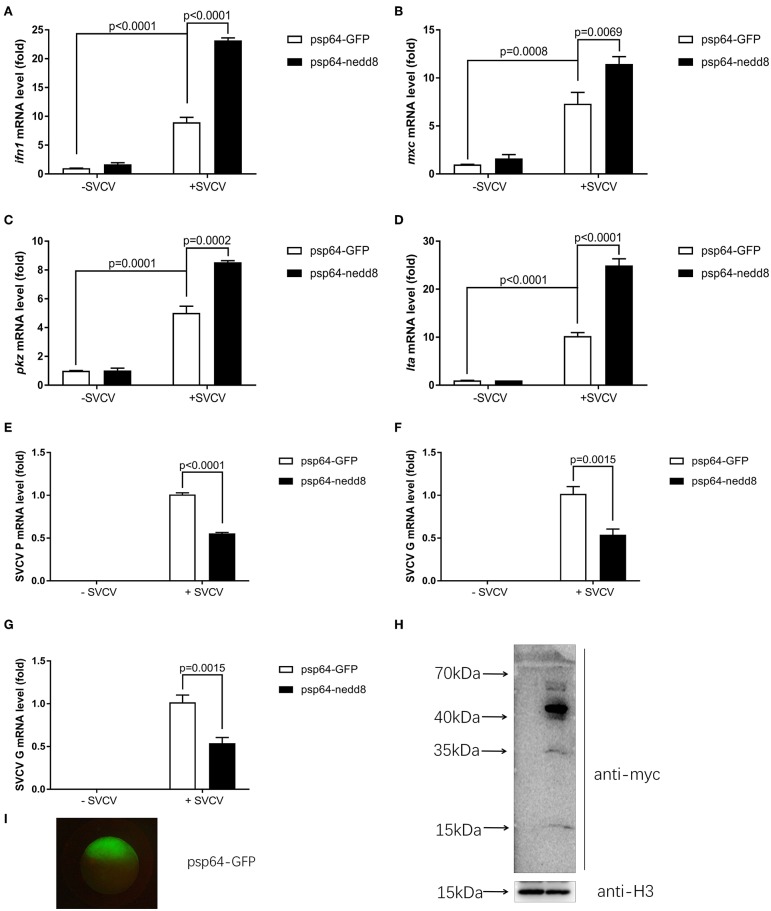
Overexpression of *nedd8* upregulates key antiviral genes after SVCV infection and suppresses viral replication *in vivo*. **(A–D)** Ectopic expression of *nedd8*, induced by mRNA injection upregulated key antiviral genes in SVCV infected zebrafish larvae. We injected zebrafish embryos at the one-cell stage with either GFP mRNA (400 pg/per embryo) or Myc-tagged *nedd8* mRNA (400 pg/per embryo). At 3 dpf, we added SVCV viruses (2 × 10^8^ TCID_50_/ml) into the water containing zebrafish larvae. After incubation for 24 h, we extracted total RNA from all larvae and performed qPCR assays to detect the expression levels of *ifn1* **(A)**, *mxc* **(B)**, and *pkz* **(C)** and *lta* **(D)**. **(E–G)** Ectopic expression of *nedd8* by mRNA injection suppressed SVCV replication in embryos. We performed qRT-PCR assays to detect the expression levels of the SVCV genes *P* **(E)**, *G* **(F)**, and *N* **(G)** genes of SVCV. **(H,I)** Western blot assay and Fluorescence micrographs of zebrafish embryos showed the expression levels of injected GFP mRNA or Myc-*nedd8* mRNA. Data are shown as mean ± SEM of three independent experiments, each performed in triplicate; the statistical analysis was performed using GraphPad Prism 5 (unpaired *t*-test).

*Nedd8* is a vital component of neddylation pathway (1, 29). Therefore, it is important to characterize the physiological functions of *nedd8*, in order to clarify the function of neddylation in various biological processes. To determine if neddylation was indeed involved in the antiviral response, we directly examined the importance of *nedd8* after viral infection. After SVCV infection, the ectopic expression of *nedd8* in zebrafish embryos via micro-injection of *nedd8* mRNA upregulated the key antiviral genes, *ifn1, mxc, pkz*, and *lta*, as compared to the embryos injected with the control (GPF) mRNA (Figures 6A–D). As expected, SVCV replication, as reflected by the expression levels of the SVCV *P, G*, and *N* genes, was suppressed in embryos injected with *nedd8* mRNA, as compared to embryos injected with GFP mRNA (Figures 6E–G). The expression levels of injected Myc-*nedd8* mRNA or GFP mRNA was confirmed by Western blot assay or fluorescent imaging (Figures 6H,I).

**Figure 7:**
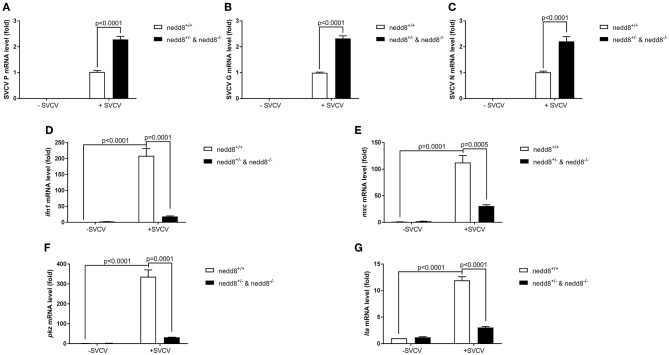
Disruption of *nedd8* in zebrafish after SVCV infection increases viral replication and downregulates key antiviral genes. **(A–C)** Viral replication increased in *nedd8*-disrupted zebrafish larvae (*nedd8*^+/−^&*nedd8*^−/−^) larvae after SVCV infection, as compared to WT larvae. **(D–G)** The expression levels of key antiviral genes after SVCV infection were lower in *nedd8*-disrupted zebrafish larvae than in WT larvae. At 3 dpf, we added viruses (2 × 10^8^ TCID_50_/ml) into the water containing zebrafish larvae. After incubation for 24 h, we extracted total RNA from all larvae and performed qPCR assays to detect the expression levels of *ifn1* **(D)**, *mxc* **(E)**, and *pkz* **(F)**, and *ita* **(G)**. Data are shown as mean ± SEM of three independent experiments, each performed in triplicate; the statistical analysis was performed using GraphPad Prism 5 (unpaired *t*-test).

We knocked out nedd8 in zebrafish via CRISPR/Cas9 (Supplemental Figure S2). Subsequently, we used *nedd8*-knockout zebrafish larvae to determine the role played by *nedd8* in the antiviral response. Due to the low fecundity of *nedd8*-null females, it was difficult to obtain pure *nedd8* ^−/−^ larvae by directly mating *nedd8* ^−/−^ (♀) with *nedd8* ^−/−^ (♂). Therefore, we mated *nedd8* ^+/−^ (♀) with *nedd8* ^−/−^ (♂) to obtain *nedd8*^+/−^*nedd8*^−/−^ larvae; we assumed *nedd8* was at least partially silenced in these larvae. Compared to the WT larvae (*nedd8*^+/+^), SVCV replication was increased in *nedd8*^+/−^*nedd8*^−/−^ larvae after SVCV infection (as reflected by the expression levels of the SVCV *P, G*, and *N* genes) (Figures 7A–C). Several key antiviral genes (i.e., *ifn1, mxc, pkz*, and *lta*) were downregulated in *nedd8*^+/−^*nedd8*^−/−^ larvae, as compared to the WT larvae, after SVCV infection (Figures 7D–G).

**Figure 8:**
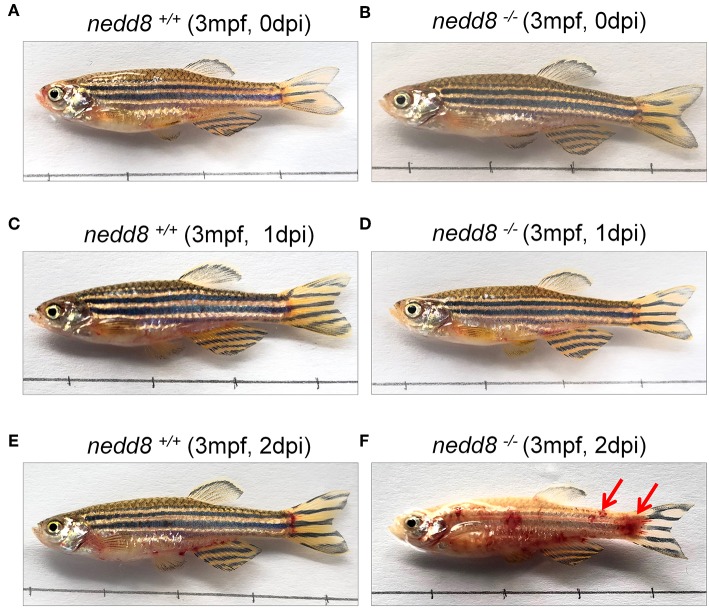
*nedd8*-null adult zebrafish are more sensitive to SVCV infection than WT zebrafish. **(A,B)** *nedd8*-null zebrafish (3 mpf; 0.38 ± 0.02 g) and the WT (3 mpf; 0.38 ± 0.02 g) were each i.p. injected with 10 μL SVCV (~2 × 10^8^ TCID_50_/ml) at 0 day. **(C,D)** At 1 day post-injection (dpi), there were no obvious differences between the WT (*nedd8*^+/+^) and *nedd8*-null zebrafish (*nedd8*^−/−^). **(E,F)** At 2 dpi, the WT zebrafish appeared normal, but the *nedd8*-null zebrafish had more swelling and hemorrhagic symptoms in the abdomen (indicated by red arrows).

**Figure 9:**
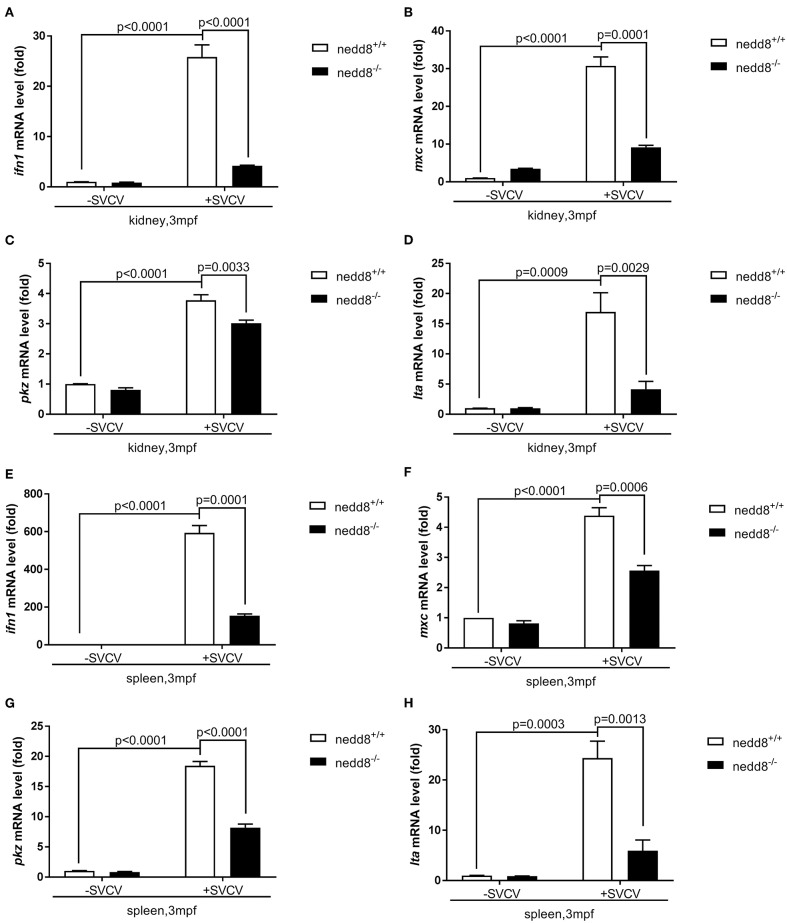
The antiviral response of *nedd8*-null adult zebrafish after SVCV infection is weaker than that of the WT zebrafish. **(A–D)** The key antiviral genes, *ifn1* **(A)**, *mxc* **(B)**, *pkz* **(C)**, and *lta* **(D)**, downregulated in the kidneys of *nedd8*-null adult zebrafish (3 mpf; 0.38 ± 0.02 g), as compared to WT zebrafish (3 mpf; 0.38 ± 0.02 g). **(E–H)** The key antiviral genes, *ifn1* **(E)**, *mxc* **(F)**, *pkz* **(G)**, and *lta* **(H)**, were downregulated in the spleens of *nedd8*^−/−^ adult zebrafish (3 mpf; 0.38 ± 0.02 g), as compared to WT zebrafish (3 mpf; 0.38 ± 0.02 g). WT (*nedd8*^+/+^) and *nedd8* ^−/−^ zebrafish were each i.p. injected with 10 μL SVCV (2 × 10^8^ TCID_50_/ml). At 2 days post-injection (dpi), we extracted total RNA from the kidneys and spleens of all zebrafish and performed qPCR assays to determine the expression levels of *ifn1, mxc, pkz*, and *lta*. Data are shown as mean ± SEM of three independent experiments, each performed in triplicate; the statistical analysis was performed using GraphPad Prism 5 (unpaired *t*-test).

To determine whether *nedd8* silencing also suppressed the antiviral response in adult zebrafish, we injected SVCV into *nedd8* ^+/+^ or *nedd8* ^−/−^ adult zebrafish (3 mpf; months post fertilization). *Nedd8*-null adult zebrafish were more sensitive to SVCV infection than WT adult zebrafish, as indicated by swelling or hemorrhage in the abdomen; and early death (Figures 8A–F). In addition, the key antiviral genes, *ifn1, mxc, pkz*, and *lta*, were downregulated in the kidneys and spleens of *nedd8*-null zebrafish, as compared to WT zebrafish (Figures 9A–H).

Thus, our results suggested that *nedd8* was essential for the antiviral response in zebrafish.

### Neddylation of Zebrafish Irf3 and Irf7 Exists

**Figure 10:**
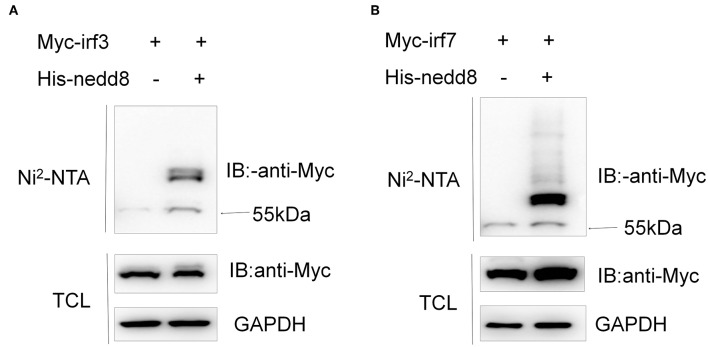
Neddylation of zebrafish Irf3 and Irf7 exists. HEK293T cells were transfected with Myc-*irf3* (5 μg) **(A)** or Myc-*irf7* (5 μg) **(B)** together with His-*nedd8* (5 μg). After 36 h, cells were lysed in guanidinium chloride, and His-*nedd8* was purified with Ni^2^-NTA agarose. TCL, total cell lysates; IP, immunoprecipitation.

To figure out the mechanisms of neddylation in zebrafish antiviral innate immunity, we conducted neddylation assays for the key factors of RLR signaling in response to viral infection (30). Neddylation of both Irf3 and Irf7 was readily detected (Figures 10A,B), but neddylation could not be detected in zebrafish Mda5, Mavs, and Tbk1 (Supplemental Figure S3). These data suggested that neddylation might facilitate antiviral response through modifying Irf3 and Irf7.

## Discussion

The role of neddylation in the host immune response to pathogenic infection has received substantial research attention (12–14, 20). However, it remains unclear whether this process negatively or positively affects the host anti-pathogen response (15, 17, 20). In this study, using both cell cultures and a zebrafish model, we showed that neddylation benefits the host during viral infection. Given that the innate immune response to viral infection is similar in zebrafish and mammals, the antiviral neddylation process might be evolutionarily conserved (22).

The innate immune system acts as the first line of defense, protecting the host from viral infection (30). Host PRRs recognize viral nucleic acids and trigger innate immune signaling cascades (30–34). These signaling cascades activate the transcription factors IRF3/IRF7 and NF-kB, inducing the antiviral response and producing IFN-1, pro-inflammatory cytokines, and other important antiviral proteins (30, 35, 36). Here, we focused on the critical genes downstream of the innate immune signaling response to viral infection in zebrafish. The inhibition of neddylation via the addition of MLN4924 or via the disruption of *nedd8* significantly downregulated these critical genes, suggesting that protein neddylation might be vital for the innate immune signaling pathway. Indeed, multiple lines of evidence indicate that post-translation modifications control innate immunity by targeting different components of the innate immune signaling pathway in various ways, including phosphorylation, ubiquitination, methylation, SUMOlation, and acetylation (37). Therefore, as an important post-translational modification, neddylation may also target components of the innate immune signaling pathway, modulating target function either directly or indirectly. Here, we identified that zebrafish Irf3 and Irf7 are possible neddylation targets. Therefore, neddylation might facilitate antiviral response through modifying Irf3 and Irf7. To further confirm neddylation Irf3 and Irf7 *in vivo* will re-enforce the importance of neddylation in antiviral innate immunity.

Based on our results, we cannot exclude the possibility that the beneficial antiviral effects conferred by neddylation were not due to modifications of innate immune signaling pathway components but instead due to modification of other molecules, such as the cullin-RING ligases.

Of note, MLN4924 can activate p53 through ribosomal-Mdm2 pathway (38). Here, we found that nedd8-deficient adult zebrafish were viable except the low fecundity displayed in *nedd8*-null females. It appears that activation of p53 resulted from deletion of nedd8 cannot affect general development other than female gonadogenesis in zebrafish. To further determine whether the defects exhibited in *nedd8*-null females are caused by p53 activation will expand our knowledge about the regulation of p53 by neddylation *in vivo*.

Increasing evidence indicates that neddylation is associated with the multiple cancer initiation and progression (11, 28, 39–41). As a potent and specific NAE inhibitor, MLN4924 has been widely used in clinical trials for cancer therapies (11, 42, 43). Here, we showed that neddylation benefits host during the antiviral response. Thus, before MLN4924; or other neddylation inhibitors are used in cancer treatments, the antiviral capabilities of patient should be carefully considered.

## Data Availability

The raw data supporting the conclusions of this manuscript will be made available by the authors, without undue reservation, to any qualified researcher.

## Author Contributions

GY and XL performed the experiments. GY, XL, and WX conceived and designed the experiments, analyzed the results, and oversaw the project. JT, CX, and GO contributed the reagents. WX wrote the main text of the manuscript. All authors reviewed and contributed to the preliminary and final draft of the manuscript.

### Conflict of Interest Statement

The authors declare that the research was conducted in the absence of any commercial or financial relationships that could be construed as a potential conflict of interest.
