# Supplementary material for: Neddylation Facilitates the Antiviral Response in Zebrafish
Source: Front Immunol. 2019 Jun 25;10:1432. doi: 10.3389/fimmu.2019.01432 (PMC6603152; doi:10.3389/fimmu.2019.01432)

Supplemental Table S1. The primer sequences (Liu et al., *J. Immunology*, 2016, 197:4736)

**Primer**	**Sequence (5' to 3')**
zebrafish *ifn1*-F	GAGCACATGAACTCGGTGAA
zebrafish *ifn1*-R	TGCGTATCTTGCCACACATT
zebrafish ifn2-F	CCTCTTTGCCAACGACAGTT
zebrafish *ifn2*-R	CGGTTCCTTGAGCTCTCATC
zebrafish *rsad*-F	AGCAGATCACCGCTCTCAAT
zebrafish *rsad*-R	CCAGACACTGGATGCTCTGA
zebrafish *mxb*-F	AATGGTGATCCGCTATCTGC
zebrafish *mxb*-R	TCTGGCGGCTCAGTAAGTTT
zebrafish *mxc*-F	GAGGCTTCACTTGGCAACTC
zebrafish *mxc*-R	TTGTTCCAATAAGGCCAAGC
zebrafish *pkz*-F	GGAGCACCGTACAGGACATT
zebrafish *pkz*-R	CTCGGGCTTTATTTGCTCTG
zebrafish *mavs*-F	GTTCCCGGTCCAAGACACTA
zebrafish *mavs*-R	TTGTCGCCTGAGTTGTTCTG
zebrafish *rig1*-F	TTGAGGAGCTGCATGAACAC
zebrafish *rig1*-R	CCGCTTGAATCTCCTCAGAC
zebrafish *lta*-F	AAGCCAAACGAAGGTCA
zebrafish *lta*-F	AACCCATTTCAGCGATTGTC
zebrafish *β-actin*-F	TACAATGAGCTCCGTGTTGC
zebrafish *β-actin*-R	ACATACAATGGCAGGGGTGTT
EPC-*ifn*-F	ATGAAAACTCAAATGTGGACGTA
EPC-*ifn*-R	GATAGTTTCCACCCATTTCCTTAA
EPC-*isg15*-F	CAGCCTTGAGGATGATTCCAG
EPC-*isg15*-R	TGCCGTTGTAAATCAGTCG
EPC-*viperin*-F	AGCGAGGCTTACGACTTCTG
EPC-*viperin*-R	GCACCAACTCTCCCAGAAAA
EPC-β2M-RT-F	CTCCATTGAACTGCTGAAAGATG
EPC-β2M-RT-R	CAAATAACTGTCTTCATTTCGCTCAT
EPC-*β-actin*-F	CACTGTGCCCATCTACGAG
EPC-*β-actin*-R	CCATCTCCTGCTCGAAGTC
SVCV-*P* protein-F	TTGGACCTGGGATAGTGA
SVCV-*P* protein-R	CTTGCTTGGTTTGTGGG
SVCV-*G* protein-F	CGACCTGGATTAGACTTG
SVCV-*G* protein-R	AATGTTCCGTTTCTCACT
SVCV-*N* protein-F	TGAGGTGAGTGCTGAGGATG
SVCV-*N* protein-R	CCATCAGCAAAGTCCGGTAT

Supplemental Figure Legends

Supplemental Figure S1. MLN4924 inhibits the expression of *ifn1* and *pkz* in zebrafish larvae upon SVCV infection.

(A-B) Zebrafish larvae (3 dpf) were pretreated with different dosage of MLN4924 for 24 h, and then were infected with SVCV (+SVCV) or PBS control (-SVCV). The expression of *ifn1* and *pkz* was examined by qRT-PCR.

Supplemental Figure S2. Generation of *nedd8*-null zebrafish via CRISPR/Cas9 technology.

(A) Scheme of the genomic structure of zebrafish *nedd8* and the sequence information in mutant *nedd8* zebrafish (mutant1: *nedd8*$^{ihb1227/ihb1227}$, in which 4 bp was deleted in exon 3). (B) The sequence information of targeting sites (the blank line) in wild type (WT) and mutant *nedd8* allele. Upper panel: the sequence of the Cas9 target site is indicated with a black line and the *nedd8* mutant allele are shown. The red dashed line indicates the deleted nucleotide. (C) The predicted protein of *nedd8* in WT and the mutant. (D) The *nedd8* mRNA level in spleens from the *nedd8*$^{+/+}$ or the *nedd8*-null mutant (*nedd8*$^{ihb1227/1227}$) (4 mpf ; n=3, respectively). (E) The Nedd8 protein in WT and the mutant larvae (3 dpf). Mpf, month post fertilization. Data are presented as means $\pm$ SEM of three independent experiments, performed in triplicate.

Supplemental Figure S3. Neddylation does not targets Mda5, Mavs and Tbk1.

(A-C) HEK 293T cells were transfected with the indicated plasmids together with His-*nedd8* respectively (5 μg/each). After 36 hr, cells were lysed in guanidinium chloride, and His-*nedd8* was purified with Ni^2-NTA agarose. TCL, total cell lysates; IP, immunoprecipitation.

Supplemental Figure S1.

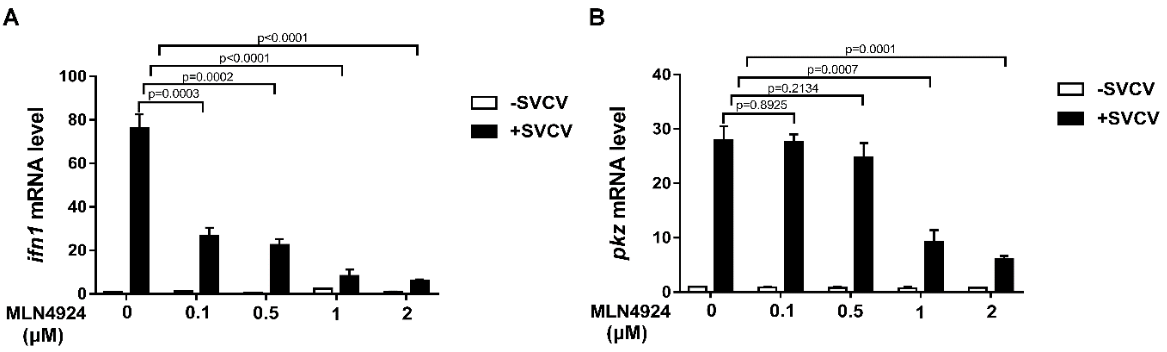

Supplemental Figure S2.

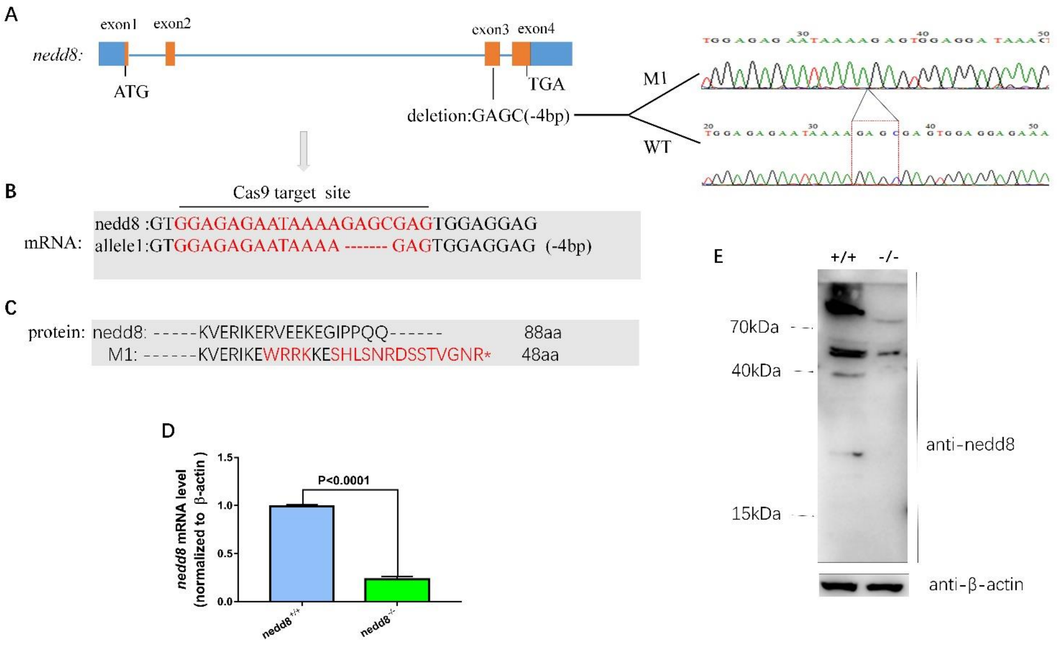

Supplemental Figure S3.

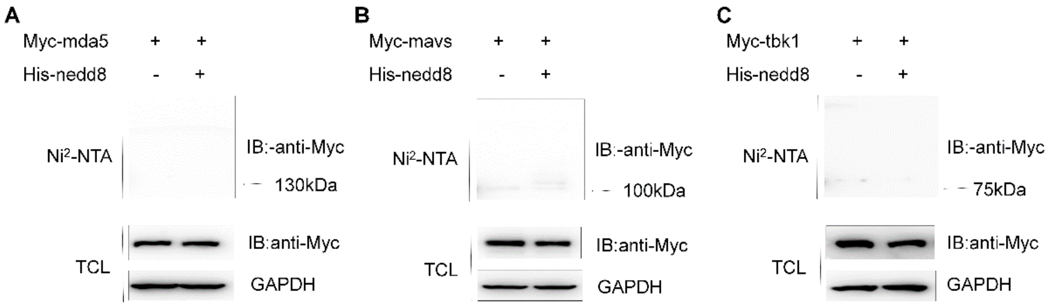

Supplement: Supplementary file 1 [file Data_Sheet_1.pdf]
